# On the hedge and safe-haven abilities of bitcoin and gold against blue economy and green finance assets during global crises: Evidence from the DCC, ADCC and GO-GARCH models

**DOI:** 10.1371/journal.pone.0317735

**Published:** 2025-02-10

**Authors:** Yasmine Snene Manzli, Mohamed Fakhfekh, Azza Béjaoui, Hind Alnafisah, Ahmed Jeribi

**Affiliations:** 1 Department of Finance, Faculty of Economics and Management of Sfax, University of Sfax, Sfax, Tunisia; 2 Department of Finance, Higher Institute of Business Administration of Sfax, University of Sfax, Sfax, Tunisia; 3 Department of Finance & Accounting, Higher School of Commerce of Tunis, University of Manouba, QuAnLab, Manouba, Tunisia; 4 Department of Economics, College of Business Administration, Princess Nourah bint Abdulrahman University, Riyadh, Saudi Arabia; 5 Department of Finance, Faculty of Economics and Management of Mahdia, University of Monastir, Monastir, Tunisia; Ovidius University of Constanta: Universitatea Ovidius din Constanta, ROMANIA

## Abstract

This paper investigates the diversification, hedging, and safe-haven capabilities of Bitcoin and gold against blue economy and green finance assets using three different MGARCH models (DCC, ADCC, and GO-GARCH) during adverse events such as the COVID-19 health crisis and the 2022 Russia-Ukraine conflict. Blue economy assets, which refer to sectors that sustainably utilize ocean resources, are a key focus alongside green finance assets. The findings reveal that during crises, Bitcoin demonstrates robust safe-haven characteristics, particularly against blue economy assets like BJLE and OCEN. Conversely, gold exhibits pronounced safe-haven properties against specific blue economy and green finance assets such as BJLE and FAN. The GO-GARCH model highlights gold’s strong diversification and safe-haven roles, especially against BJLE. Bitcoin, on the other hand, is more effective as a diversifier for PIO. Moreover, the GO-GARCH model consistently outperforms the DCC and ADCC models in terms of hedging effectiveness, showing that gold is the preferred hedging instrument for GNR and TAN, while Bitcoin is more effective for other blue and green assets. The results underscore the distinct roles of Bitcoin and gold in portfolio management strategies, offering insights for investors navigating market uncertainties in the context of sustainable investments.

## 1. Introduction

Following the Global Financial Crisis (GFC) in 2008, investors have explored alternative avenues to spread their investments across different asset markets to manage the trade-off between risk and return [1]. Nevertheless, the process of globalization has resulted in interconnectedness and associations among these markets [2]. Scholars have examined how spillovers affect conventional asset markets and the interconnectedness of stock markets in both advanced and developing economies [3–6]. As Industry 4.0 gains prominence and multiple climate change problems rise, attention has shifted towards emerging asset markets such as blue economy and green finance assets [7–11].

Climate change and sustainable markets have emerged as significant issues for numerous governments, businesses, households, policymakers, and academic communities in recent years [12]. For example, in the United States, the value of sustainable and eco-friendly investment assets soared to $12 trillion in 2018 (US SIF, 2018). This indicates that such assets have expanded almost 18 times since 1995 and have grown by 38% since 2016 [13]. These facts demonstrate that environmental and sustainability issues have influenced eco-friendly financing and investment approaches. Furthermore, the concepts of the blue economy and green finance assets are becoming increasingly popular, highlighting the sustainable utilization of marine resources and environmentally responsible financial practices.

Blue economy assets include a wide array of economic activities connected to the sustainable use of ocean resources, promoting economic development, enhancing livelihoods, and supporting ecosystem health [9]. These assets encompass marine commerce, offshore energy generation, fisheries, aquaculture, renewable ocean energy, coastal tourism, and maritime transport [14]. On the other hand, green finance assets encompass various financial products and services, such as green bonds, green investment funds, carbon markets, and energy trading. It also involves innovative financial institutions like green banks and green funds [15]. Therefore, to mitigate the perilous global warming of the past decade, it is essential to swiftly and sufficiently invest in renewable energies and increase investments in blue and green financial assets [16–19-19]. Specifically, [20] discover that investors have redirected their attention toward environmentally conscious investments in recent years and now incorporate sustainable companies when constructing portfolios. [21] highlight that green bonds decrease worldwide coal usage, consequently raising the proportion of non-fossil electricity, presenting a method to further diminish global CO2 emissions. [22] underscores the significance of evaluating the sustainability of blue economy endeavors by assigning value to the marine ecosystem. [23] reveal that blue bonds provide strong financial returns and offer substantial environmental and social effect on ocean economies.

From an academic standpoint, many researchers have increasingly struggled to study the relationship between green bonds and other asset classes. For instance, [24] analyze the comovements among green bonds and other asset classes (commodities, stocks, clean energy, and conventional bonds). The authors show the dynamic characteristics of correlation among asset pairs over time. [25] analyze the time-frequency connectedness between green bond markets and financial and energy markets to test if green bonds are a distinct asset class. Their findings reveal that connectedness primarily occurs at shorter time horizons, indicating that shocks are transmitted between different markets. High connectedness in volatility and return is observed between green bonds and Treasury and investment-grade corporate bonds, leading them to conclude that green bonds are not a distinct asset class. [11] study the time-varying spillovers between green finance markets and the effects of investor sentiment and climate policy uncertainty on these spillovers. The authors find that green bond, carbon, green building, and green transportation markets are information receivers, while water, clean energy, and environmental, social, and governance (ESG) markets are information transmitters. Post-COVID-19, the green bond market appears more interconnected with other green finance markets. The authors also show that investor sentiment increases the net total directional spillovers of green markets. [26] report that the spillover effect of the Russia-Ukraine conflict on the green finance market is moderate, whereas the health crisis significantly affects the spillover of both higher- and lower-order moments in this market.

It is important to note that this geopolitical conflict has exacerbated the need for investors to seek safe-haven assets [6,27]. The Russia-Ukraine war, along with other crises like the COVID-19 pandemic, has disrupted global financial markets, causing heightened inflation and uncertainties regarding energy supply, particularly from Russia [27]. These disruptions make the exploration of safe-haven assets in portfolios that include environmentally-focused investments, like blue economy and green finance assets, even more critical. The war has also underscored the importance of diversifying across non-traditional assets to hedge against market volatility and geopolitical risks.

Against this backdrop, it is crucial to investigate the relationship between blue economy and green finance assets, as well as other asset classes, to understand better the dynamic connectedness and co-movement of different assets within a portfolio. Furthermore, given the vital role these assets play in economic growth and environmental sustainability, it is essential to identify and manage the risks associated with investing in the blue economy and green finance assets, particularly in light of the recent crises related to the COVID-19 pandemic and the Russia-Ukraine conflict. In this context, the search for effective safe-haven assets becomes crucial for risk-averse investors [28]. The adverse consequences of health and political crises have increasingly drawn investors’ attention to assets that can minimize portfolio risk, reviving interest in various assets’ hedging, diversification, and safe-haven features. From an academic standpoint, many researchers have investigated these features in gold and cryptocurrencies [6].

Gold has a well-established reputation as a store of value and a dependable safe haven asset during periods of instability [6,28–34]. On the other hand, Bitcoin, a relatively recent addition to the safe haven category, has shown promise as a digital alternative. It has attracted the attention of many investors as a hedge against stock market downturns [6,32,35–40]. While previous studies have explored the safe-haven characteristics of gold and Bitcoin separately [28,29], and sometimes, in comparison to specific assets and commodities [6,27,32,39–42] or during specific crises [6,27,39,43,44], there remains a gap in understanding how these assets perform as safe havens against environmental assets. In this regard, our study aims to address this literature gap by analyzing the correlation framework and the diversification benefits of gold and Bitcoin in comparison to blue and green assets. Specifically, our research seeks to examine and evaluate the hedging and safe-haven characteristics of gold and Bitcoin against blue economy and green finance assets, particularly in the wake of unexpected events such as the COVID-19 pandemic and the Russia-Ukraine crisis.

In spite of the ongoing course to find the best alternative asset in terms of risk management and portfolio diversification, there is no ubiquitous consensus among researchers who use different countries, sectors and periods. In this respect, several studies have particularly focused on the safe-haven, diversification and hedging capabilities of Bitcoin and gold against other assets such as stocks, commodities and cryptocurrencies. Other studies have rather attempted to compare the characteristics of gold with those of Bitcoin [e.g., 45]. For instance, [45] attempt the outperformance of gold as a safe-haven compared to Bitcoin. They show that gold seems to a safe-haven asset for crude oil and stock markets during the COVID-19 pandemic. Compared to Bitcoin, the safe-haven performance of gold against the stock markets has improved during times of high spread of the pandemic health. From the foregoing, this study is motivated by the presence of several inconsistent results concerning the linkages among Bitcoin, gold and other assets and their potential diversification advantages. More specifically, the idea behind this study is to analyze the safe-haven, diversifying and hedging capabilities of gold and Bitcoin against blue economy and green assets during normal and turbulent times. To this end, we use the three different MGARCH models (DCC, ADCC and GO-GARCH). Using such different econometric models allow us to learn much information about different aspects of behavior of correlation and volatility. We thereafter perform the hedging effectiveness in order to compare between such econometric models and examine how hedge ratios differ across GARCH models. Such models allow us to model and estimate in a more adequate fashion the volatility dynamics, conditional correlations and hedge rations between Bitcoin/gold and blue economy/green finance assets. Specifically, our study aims to address the following research questions:

- How effective are gold and Bitcoin as safe-haven assets during periods of crisis for blue economy and green finance assets?
- Which model outperforms the other ones in terms of risk reduction?

The results of our study reveal that gold outperforms Bitcoin as a diversifier and a safe-haven asset during turbulent times. This emphasizes its potential as a comparatively stable and protective asset in comparison with Bitcoin, supporting the findings of [46,47], who show that gold outperforms Bitcoin as a safe-haven asset during turbulent times. The hedge ratios and hedging effectiveness results show that gold is the best hedging instrument for the blue economy asset SPDR S&P Global Natural Resources ETF (GNR) and the green finance asset Invesco Solar ETF (TAN), while Bitcoin is more effective for other blue economy and green finance assets. Bitcoin generally offers lower hedge ratios compared to gold, indicating a more cost-effective hedge. However, gold’s hedging effectiveness is superior when considering volatility spillover, making it a more robust option during turbulent periods.

Our paper makes substantial contributions to the existing literature on cross-asset holdings in multiple dimensions. First, unlike previous studies that have primarily focused on green bonds [19,26,48], we expand the scope by analyzing the correlation structure between blue economy and green finance assets, alongside gold and Bitcoin. This broader analysis provides comprehensive insights into the interconnectedness and relationship dynamics among a diverse set of asset classes. Second, while prior research has extensively examined the hedging, diversification, and safe-haven features of green bonds against other asset classes [19,49–53], our study breaks new ground by being the first to explore the potential of hedging blue economy and green finance assets with gold and Bitcoin. We delve into the hedging, diversification, and safe-haven characteristics of these assets in the face of significant adverse events, such as the COVID-19 pandemic and the Russia-Ukraine conflict. Employing three advanced econometric models—DCC, ADCC, and GO-GARCH—we offer a robust and nuanced analysis that deepens the understanding of asset behavior and risk management strategies during periods of crisis. Additionally, we assess the performance of each model to identify the most effective approach.

The remainder of this paper is as follows. Section 2 presents the literature review. Section 3 presents the methodology. Section 4 describes the data. Section 4 presents the estimation results and discussion. Finally, Section 6 concludes.

## 2. Literature review

Broadly speaking, the advent of unexpected and unprecedented events such as the COVID-19 pandemic and the Russia-Ukraine conflict have increasingly caused severely adverse consequences on the global economy and raised the investment risk in international financial markets. Numerous analysts and researchers have associated the decline in global financial markets with the ongoing COVID-19 pandemic [54–56], which has resulted in volatile and negative overall market responses. [54] report that the spread of COVID-19 is a significant shock to global economies, resulting in a drastic decline in the stock market. [55] report that the effects of the COVID-19 pandemic could be highly detrimental to investors and the global economy. [57,58] state that the recent COVID-19 health crisis has affected all global financial markets, notably causing a substantial and ongoing decline in stock prices. [59] also indicate that COVID-19 has a significantly adverse effect on market indices.

Moreover, it is necessary to stress the importance of the Russia-Ukraine war. This conflict has not only contributed to significant market volatility but also highlighted the fragility of global supply chains, especially for energy resources. Studies like [60] emphasize how disruptions in Russian energy supply have raised inflation risks and slowed down global growth. The war’s impact on commodity prices, particularly energy and agricultural goods, has increased volatility across asset markets, necessitating a stronger focus on assets that can hedge against such geopolitical shocks [27]. This adds to the growing body of literature that stresses the critical role of safe-haven assets during times of geopolitical conflict.

In fact, the Russia-Ukraine war has led to ripple effects, notably causing significant turmoil and an unprecedented rise in the risk of global financial markets [61,62]. The Russia-Ukraine crisis leads to slow global growth and raise inflation as global growth risk is linked to Russia’s energy supply disruption [60]. The first War day wiped nearly $1 trillion off the value of the global stock market. This war accelerated a drop in the major financial indices as international investors have started to get jittery about major central bank rate hikes to battle inflation [63]. In addition, gold set for the biggest quarterly gain in nearly two years on the Russia-Ukraine conflict, leading to inflation fears.

Unlike prior economic and financial downturns, the forces behind these recent crises have introduced a range of challenges and risks, prompting investors to seek uncorrelated assets to protect their stock market portfolios [5,6,27,64,45], particularly diversifying, hedging, and safe haven assets. In fact, [28] were the first to define hedging, diversifying, and safe-haven assets. According to these authors, hedging assets protect portfolios during normal times, while diversifying assets reduce portfolio risk and enhance diversification in both normal and stressful periods. Safe-haven assets, on the other hand, safeguard portfolios during economic uncertainty, market volatility, or geopolitical turmoil.

Gold is the asset most commonly discussed in literature concerning hedging and seeking a safe haven during times of crises. Its lack of correlation with other financial assets makes it an attractive choice for investors aiming to broaden their stock market portfolios and shield against market volatility [6,29,27,65–67]. [68] examine the function of gold as a hedge and safe haven against equity market indices of leading gold-producing nations. Their findings, based on the GARCH model, underscore gold’s capacity as a hedge and safe haven across various segments of the return distribution. Using a GED-GARCH model, [69] examines the hedging and safe haven properties of gold across 10 countries. The author’s findings suggest that during scenarios of stock market downturns, inflation, currency depreciation, and economic policy uncertainty, gold acts as both a hedge and safe haven across all examined countries. [46,47] show that gold outperforms Bitcoin as a safe-haven asset during turbulent times.

Employing an innovative Quantile-VAR interconnectedness methodology, [6] explore the hedging and safe haven abilities of gold and Bitcoin against the G7 stock market indices amid the COVID-19 pandemic, the Russia-Ukraine military conflict, and the collapse of the Silicon Valley Bank. Their findings indicate that both gold and Bitcoin serve as efficient hedges in normal market circumstances and robust safe-haven assets during the three crises. The authors also state that gold stands out as the predominant safe haven asset, surpassing Bitcoin, particularly during the conflict and the SVB collapse. Moreover, during the same three crisis periods, [27] investigate the hedging and safe haven abilities of gold and Bitcoin against energy and agricultural commodities. Their findings also indicate that gold outperforms Bitcoin as a diversifier and a safe haven for commodities.

[70] explores whether gold can function as a hedge or safe-haven asset for the U.S. dollar’s exchange rate risk against a diverse array of currencies. The results suggest the significant role of gold in hedging exchange rate risks. Additionally, gold serves as a safe-haven asset during the COVID-19 pandemic and the Russia-Ukraine conflict for certain exchange rate pairs analyzed. [71] explore the correlation between energy metals and precious metals to evaluate their appropriateness as safe-haven assets within clean energy investment portfolios. Their results affirm the dependability of precious metals, including gold, as safe-haven assets for clean energy stock indices. [72] examine the diversification, hedging, and safe haven characteristics of gold for the G7 stock market indices across varying market situations. Their findings validate gold’s diversification ability for the G7 stock markets under all market conditions.

[73] explore the hedging and safe-haven features of gold and other precious metals, along with three major indices in the U.S. market. Their findings reveal that metal markets are recognized for their hedging properties during periods of financial distress. [74] study and compare the safe-haven characteristics of gold, Bitcoin, and a gold-backed cryptocurrency against the stock and banking indices of G7 nations during periods of adverse events. They demonstrate that gold and a gold-backed cryptocurrency are perfect safe havens when compared to Bitcoin. [75] investigate the role of gold as a hedge and safe haven from the perspective of Chinese investors. Their findings indicate that gold is a safe haven during the two crash periods analyzed. [76] investigate the safe-haven capabilities of gold amidst the COVID-19 pandemic. Their findings validate gold’s ability as a safe haven during the pandemic, although its effectiveness was higher before the pandemic. Additionally, their findings indicate that gold consistently demonstrates superior safe haven characteristics compared to US stocks and other precious metals regardless of the timeframe.

Aside from the precious metal, digital currencies have also garnered the interest of investors as hedging and safe haven instruments [77]. For example, Bitcoin, distinguished by its absence of correlation with traditional assets [35] and its autonomy from the monetary policy environment [78], possesses the ability to reduce portfolio risk and provide hedging benefits amid periods of financial market turmoil [6,47,79–81].

[82] evaluate cryptocurrencies as hedging tools, emphasizing Bitcoin’s efficacy in hedging amid the COVID-19 pandemic. [83] show that Bitcoin investment patterns can usually be predicted during stock market downturns, suggesting its hedging utility. Using the DVECH-GARCH model, [84] evaluate Bitcoin’s ability to hedge against G7 stock indices during the COVID-19 pandemic and the Russia-Ukraine conflict. Their results reveal that before the COVID-19 outbreak, Bitcoin was a proficient hedging instrument. However, during the pandemic and the conflict, it demonstrates diversification characteristics. The research also suggests that Bitcoin may be viewed as a safe-haven asset. Using the DCC-GARCH model, [85] examine the hedging and safe haven properties of Bitcoin and gold during the recent crises of the COVID-19 pandemic and the Russia-Ukraine conflict. Their findings suggest a similarity in hedging abilities between gold and Bitcoin. Specifically, both have shown limited safe-haven characteristics during the COVID-19 health crisis and robust safe-haven characteristics during the Russian-Ukrainian conflict.

[72] state that cryptocurrencies, including Bitcoin, possess significant safe-haven potential for the G7 stock market indices, especially during the COVID-19 pandemic. [86] employ DCC–ARMA–GARCH models to analyze the diversification, hedging, and safe-haven attributes of Bitcoin and Ethereum across different financial assets. Their results indicate that cryptocurrencies fulfill these distinct functions across particular asset markets during varying timeframes. [87] examine Bitcoin’s safe-haven and hedging characteristics across a broad range of traditional assets both prior to and amid the COVID-19 pandemic. Their findings indicate that Bitcoin exhibits safe-haven properties during the COVID-19 pandemic, whereas it acts as a hedging instrument in the period before the pandemic.

Using the multivariate ADCC model, [88] examines the hedge and safe haven properties of cryptocurrencies [Bitcoin and Ethereum] against major global traditional currencies during the COVID-19 outbreak and the Russia-Ukraine military conflict. They indicate that cryptocurrencies serve as safe havens for nearly all traditional currencies. [89] investigate the safe haven properties of Bitcoin during the Silicon Valley bank collapse. Their findings support Bitcoin’s safe haven capability, indicating that it outperformed gold and other traditional assets during this crisis. [90] examine the hedging and safe-haven characteristics of Bitcoin and other financial assets across ten countries most impacted by the COVID-19 pandemic: the USA, Brazil, the UK, Italy, Spain, Germany, France, Russia, China, and Malaysia. Their findings indicate that Bitcoin serves as a robust hedge in the USA and as both a strong hedge and safe haven in China.

Based on the above discussion, gold and Bitcoin demonstrate significant effectiveness in diversifying, hedging, and acting as safe-haven assets across various traditional financial assets and during different crises. However, the literature currently lacks examination of these attributes in relation to emerging assets like blue economy and green finance. Our study aims to address this gap in the literature.

## 3. Methodology

To reduce a portfolio risk including blue/green indices and Bitcoin/Gold, it is worth noting to compute the optimal hedging ratio. That is why one might estimate conditional variances and the conditional correlation using three Multivariate Generalized Autoregressive Conditional Heteroskedasticity (MGARCH) models (DCC, ADCC, GO-GARCH). We afterwards perform the hedging effectiveness to compare between different models with the aim of examining the extent to which hedge ratios differ across GARCH models.

### 3.1. The dynamic conditional correlation (DCC) model

The dynamic conditional correlation (DCC) model allows the conditional correlation matrix to vary over time. Developed initially by [91], the DCC model is estimated in two-step estimation procedure. We first estimate the GARCH model parameters and we afterwards perform the time-varying correlations. Accordingly, the DCC model is presented as follows:

The return equation:


$$r_{i,t}=\mu_{i,t}+ar_{t-1}+\varepsilon_{i,t}$$ (1)

The conditional variance equation with p=q=1:


$$h_{iit}=\omega_{i}+\alpha_{i}\varepsilon_{i,t-1}{}^{2}+\beta_{i}h_{ii,t-1}$$ (2)

ω>0, α>0 et β>0

The DCC-GARCH model is presented as follows:


$$H_{t}=D_{t}R_{t}D_{t}$$ (3)

where:

−*H_t_* is the 2  ×  2 conditional covariance matrix;

- $R_{t}$ refers to the conditional correlation matrix; and

- $D_{t}$ denotes a diagonal matrix with time-varying standard deviations.


$$D_{t}=\text{diag}\left(\sqrt{{\text{h}}_{11}},\;\sqrt{{\text{h}}_{22}}\right)$$ (4)

$$D_{t}=\left(\begin{array}{ccccc} h_{1,t}{}^{\frac{1}{2}} & 0 & 0 & \cdots & 0\\ 0 & h_{2,t}{}^{\frac{1}{2}} & 0 & \cdots & 0\\ 0 & 0 & h_{3,t}{}^{\frac{1}{2}} & \cdots & 0\\ \vdots & \vdots & \vdots & \ddots & \vdots \\ 0 & 0 & \cdots & \cdots & h_{N,t}{}^{\frac{1}{2}} \end{array}\right)$$

and


$$R_{t}=diag({\left(Q\right)}^{-\frac{1}{2}})Q_{t}diag({\left(Q\right)}^{-\frac{1}{2}})$$ (5)

with Qt corresponds to a (2  ×  2) symmetric positive definite matrix $Q_{t}=q_{t}^{ij}$, and is given as:


$$Q_{t}=\left(1-\theta_{1}-\theta_{2}\right)\bar{Q}+\theta_{1}\varepsilon_{t-1}\varepsilon {'}_{t-1}+\theta_{2}Q_{t-1}$$ (6)

where $\bar{Q}$ is a (2  ×  2) matrix of the unconditional correlation of standardized residuals; $\theta_{1}$ and $\theta_{2}$ are non-negative scalars, assuming that $\theta_{1}+\theta_{2}<1$. The correlation estimates are given as follows:


$$\rho_{i,j,t}=\frac{q_{i,j,t}}{\sqrt{q_{ii,t}.q_{jj,t}}}$$ (7)

### 3.2. The asymmetric dynamic conditional correlation (ADCC) model

[92] developed the DCC model and asymmetric GARCH model proposed by [93]. They perform the following asymmetric DCC [ADCC] model by including an asymmetric term:


$$h_{i,t}=\omega_{i}+\alpha_{i}\varepsilon_{i,t-1}{}^{2}+\beta_{i}h_{ii,t-1}+d_{i}\varepsilon_{i,t-1}{}^{2}I\left(\varepsilon_{i,t-1}\right)$$ (8)

ω>0, α>0 and β>0

where *h*_*t*_ refers to the conditional variance; $\omega_{i}$ corresponds to a constant; $\alpha_{i}$ and $\beta_{i}$ are the parameters which capture the persistence of short- and long-term volatilities, respectively; and ${\text{d}}_{i}$ corresponds to the asymmetric parameter.

The indicator function $I\left(\varepsilon_{i,t-1}\right)\;$ is equal to one if $\varepsilon_{i,t-1}<0$, and 0 otherwise. In this respect, a positive value of d reflects the existence of the negative residuals, rather than the positive ones. This afterwards leads to increase variance. The asymmetric effect (the so-called “leverage effect”) captures the fact that an unexpected decrease in asset prices tends to increase volatility more than an unexpected increase with the same magnitude. This also implies that bad news tend to contribute in increasing volatility more than the good ones.

Based on the ADCC model, the Q dynamics are given as follows:


$$Q_{t}=\left(\bar{Q}-A{'}^{\bar{Q}}A-B{'}^{\bar{Q}}B-G{'}^{{\bar{Q}}^{-}}G\right)+A{'}^{z_{t-1}z_{t-1}^{'}}A+B{'}^{Q_{t-1}}B+{G^{\prime }}^{z_{t}^{-}z_{t}^{'-}}G$$ (9)

In Eq.(9), *A*, *B* and *G* are n×n parameter matrices, and $z_{t}^{-}$ are zero-threshold standardized errors that are equal to $z_{t}$ which tend to be inferior to 0, and 0 otherwise. $\bar{Q}$ and ${\bar{Q}}^{-}$ are the unconditional matrices of $z_{t}$ and $z_{t}^{-}$, respectively.

### 3.3. The generalized orthogonal GARCH (GO-GARCH) model

[94] propose the generalized orthogonal GARCH model by assuming that asset returns $r_{t}$ follow this process:


$$r_{t}=n_{t}+\varepsilon_{t}$$ (10)

where: $n_{t}$ refers to the conditional mean and rep $\varepsilon_{t}$ resents the error term.

The GO-GARCH model includes $r_{t}-n_{t}$ using a set of unobserved exogenous factors, as follows:


$$\varepsilon_{t}=Bf_{t}$$ (11)

where *B* represents a mixing matrix that is disintegrated in an orthogonal matrix *R* and an unconditional covariance matrix *Π*, such as:


$$B=\Pi^{1/2}R$$ (12)

Herein, the rows in the mixing matrix B denote the assets whereas the columns include factors which are given as follows:


$$f_{t}=G^{1/2}z_{t}$$ (13)

where the random variable $z_{t}$ is characterized as $E\left(z_{it}\right)=0$ and $E\left(z_{it}^{2}\right)=1$.

One might specify the factor conditional variances using a GARCH model. So, one might combine Eqs. (10) (11) and (14) as follows:


$$r_{t}=n_{t}+BG_{t}^{1/2}z_{t}$$ (14)

One might also specify the conditional covariance matrix of asset returns, $r_{t}-n_{t}$, as follows:


$$\Pi_{t}=BG_{t}B^{\prime }$$ (15)

It is worth noting that the GO-GARCH model takes into consideration the two following assumptions: *B* is time invariant and the $G_{t}$ matrix is diagonal. According to [94], one might use a single-step maximum likelihood method in order to estimate concurrently the orthogonal matrix with the adequate dynamics. Nevertheless, such method seems to be hard to use when prevalent multiple assets exist. The orthogonal matrix R has been recently proposed to perform estimations through independent component analysis. A similar approach has been used in this paper.

### 3.4. Testing the hedging and safe-haven properties of gold and bitcoin

The hedging, safe-haven and diversification features of particular financial assets arise from its overall correlation with other assets. Following [29,95,96], we estimate the following model to examine such properties for Bitcoin and Gold against blue and green assets:


$$\rho_{t}=\gamma_{0}+\gamma_{1}D\left(r_{BL}q_{1}\right)+\gamma_{2}D\left(r_{BL}q_{5}\right)+\gamma_{3}D\left(r_{BL}q_{10}\right)+\varepsilon_{t}$$ (16)

where $\rho_{t}$ is the dynamic conditional correlation between Bitcoin/Gold and each blue/green asset. $\gamma_{0}$ is a constant; $\gamma_{1}$, $\gamma_{2}$, $\gamma_{3}$ are the coefficients related to the extreme movements. $r_{BL}q_{1}$; $r_{BL}q_{5}$; $r_{BL}q_{10}$ are the rankings of the asset return series at 1%, 5%, and 10%, respectively; and the error term refers to $\varepsilon_{t}$:

We estimate Eq.(16) using the three Multivariate GARCH models during the whole period which is characterized by the outbreak of the COVID-19 pandemic and the Russia–Ukraine war. Based on the results of Eq.(6), the diversification, hedging, and safe-haven features are apprehended as follows:

- Bitcoin/Gold is a strong (resp. weak) hedge if $\gamma_{0}$ is negative (resp. zero).
- If $\gamma_{0}$ is positive but not strongly correlated, Bitcoin/Gold can be considered as a diversifier.
- Bitcoin/Gold is a strong (resp. weak) safe-haven if $\gamma_{1}$, $\gamma_{2}$ or $\gamma_{3}$ are negative (resp. insignificantly different from 0).

The model expressed in Eq.(16) examines the hedging and safe-haven characteristics of Bitcoin and Gold during the turbulent periods of crises implicitly and statistically as we consider three percentiles.

### 3.5. Hedge ratio and hedging effectiveness

To compute the optimal hedge ratio, we need to estimate the conditional variance and covariance estimates given that it highly depends on minimizing the portfolio return variance [97]. The risk-minimizing hedge ratio between asset i and asset j is given as follows:


$$\beta_{\text{ij},\text{t}}=\frac{{\text{h}}_{\text{ij},\text{t}}}{{\text{h}}_{\text{jj},\text{t}}}$$ (17)

where h_ij,t_ refers to the conditional covariance between asset i and *j* at time *t*, ${\text{h}}_{\text{jj},\text{t}}$ is the conditional variance of asset *j* at time *t* Herein, a long position in one Dollar in asset i (i.e., buying asset i) can be hedged by a short position in $\beta_{\text{ij},\text{t}}$ Dollars of asset *j* (i.e., selling asset *j*).

Afterwards, we calculate the hedging effectiveness (HE) for each portfolio composed by Bitcoin/Gold and blue/green asset. As recommended by [98], this can be determined by analyzing the achieved hedging errors. The HE index is given as follows:


$$HE=\frac{Var_{unhedged}-Var_{hedged}}{Var_{unhedged}}$$ (18)

where $Var_{hedged}$ corresponds to the returns’ variance of the Bitcoin/Gold-blue/green asset portfolio, and $Var_{unhedged}$ indicates the variance of blue/green assets. A greater HE suggests a higher portfolio risk reduction. Therefore, the risk-minimization strategy could be considered as an effective hedging strategy.

## 4. Data and descriptive statistics

### 4.1. Data

In this study, we analyze four blue economy assets, namely BNP Paribas Easy ECPI Global ESG Blue Economy UCITS ETF [BJLE], IQ Clean Oceans ETF (OCEN), SPDR S&P Global Natural Resources ETF (GNR), and Invesco Global Water ETF (PIO). Additionally, we consider four green assets, which include SPDR S&P Kensho Clean Power ETF (CNRG), iShares Global Clean Energy ETF (ICLN), Invesco Solar ETF (TAN), and First Trust Global Wind Energy ETF (FAN). Moreover, we incorporate the daily prices of Bitcoin and Gold. The data used in this study are global in nature, representing a wide range of geographic markets and sectors within the blue and green economies. Our dataset encompasses a total of 448 observations, spanning the period from October 25, 2021, to January 5, 2024. This period is marked by significant adverse events such as the COVID-19 pandemic and the Russia-Ukraine war. The data were sourced from Datastream. Detailed descriptions of these assets, including their full names, relevance to the blue and green economy, and measurement descriptions, are provided in Table 1.

**Table 1:** Description of Blue Economy and Green Finance assets.

Category	Ticker	Full Name	Description	Measurement Description
**Blue Economy assets**	BJLE	BNP Paribas Easy ECPI Global ESG Blue Economy UCITS ETF	Invests in companies contributing to the blue economy, focusing on sustainable use of ocean resources.	Daily closing prices, sourced from Datastream
	OCEN	IQ Clean Oceans ETF	Targets companies involved in reducing ocean pollution and promoting marine conservation.	Daily closing prices, sourced from Datastream
	GNR	SPDR S&P Global Natural Resources ETF	Includes firms in the natural resources sector, many of which focus on sustainable and renewable resources.	Daily closing prices, sourced from Datastream
	PIO	Invesco Global Water ETF	Focuses on companies engaged in water conservation and management, critical components of the blue economy.	Daily closing prices, sourced from Datastream
**Green Finance assets**	CNRG	SPDR S&P Kensho Clean Power ETF	Invests in companies involved in clean and renewable energy production.	Daily closing prices, sourced from Datastream
	ICLN	iShares Global Clean Energy ETF	Focuses on global clean energy firms, promoting sustainable energy solutions.	Daily closing prices, sourced from Datastream
	TAN	Invesco Solar ETF	Targets companies that generate energy from solar power, a key green economy sector.	Daily closing prices, sourced from Datastream
	FAN	First Trust Global Wind Energy ETF	Includes firms engaged in wind energy production, another critical area of the green economy.	Daily closing prices, sourced from Datastream
**Other Assets**	BTC	Bitcoin	A cryptocurrency often considered a digital store of value and investment asset.	Daily closing prices, sourced from Datastream
	Gold	Gold	A traditional commodity used as a store of value and investment hedge.	Daily closing prices, sourced from Datastream

We acknowledge that the selected period does not include a non-crisis reference period. However, the data for these specific assets (blue economy and green finance assets) are only available starting from October 25, 2021. Our primary aim is to analyze the performance of blue economy and green finance assets specifically under adverse conditions. By focusing on this crisis period, we can provide insights into the resilience and behavior of these assets during times of global uncertainty and economic stress. Understanding asset performance in such conditions is crucial for investors and policymakers interested in sustainable investments and their stability in the face of significant disruptions.

All the prices are converted into daily returns as follows:


$$r_{it}=log\left(\frac{p_{i,t}}{p_{i,t-1}}\right)$$

where $p_{i,t}$ $p_{i,t-1})\;$ represents the closing price of asset i at time t (t-1).

### 4.2. Descriptive statistics

The descriptive statistics for the return series are presented in Table 2. Gold shows the highest mean return (0.013), whereas Bitcoin reports a negative mean return of −0.170. All blue economy and green assets exhibit negative mean values, except for GNR, which records a positive mean return (0.004). Bitcoin appears to be the most volatile asset, given its highest standard deviation value (3.808).

**Table 2:** Descriptive statistics.

	Mean	Median	Max.	Min.	Std.Dev.	Skewness	Kurtosis	Jarque-Bera	Q[12]	Q^2^[12]	LM	Obs.
**BJLE**	−0,005	0,069	3,410	−3,267	0,992	−0,128	3,709	10,615***	14,402	47,425***	31,421***	448
**OCEN**	−0,043	−0,047	7,077	−4,122	1,396	0,313	4,343	40,976***	11,679	47,636***	28,171***	448
**GNR**	0,004	0,026	4,419	−6,092	1,553	−0,301	3,835	19,800***	13,037	72,976***	45,656***	448
**PIO**	−0,032	0,000	5,826	−4,156	1,318	0,163	3,920	17,790***	11,093	50,437***	28,354***	448
**ICLN**	−0,078	−0,219	7,350	−6,163	1,952	0,451	4,265	45,067***	19,507 *	39,235***	28,079***	448
**CNRG**	−0,065	−0,122	8,206	−5,989	2,196	0,284	3,432	9,504***	10,263	15,339	19,787 *	448
**FAN**	−0,066	−0,058	6,369	−4,333	1,440	0,370	4,575	56,526***	21,282**	47,343***	33,436***	448
**TAN**	−0,086	−0,281	8,907	−7,800	2,640	0,355	3,777	20,644***	13,569	37,426***	30,039***	448
**BITCOIN**	−0,170	−0,151	18,120	−28,683	3,808	−0,950	12,328	1691,410***	2,660	5,518	19,974 *	448
GOLD	0,013	0,038	3,402	−2,836	0,906	0,065	3,817	12,769***	20,100 *	23,178**	19,476 *	448

The skewness coefficients indicate that most assets’ returns exhibit a rightward skew, evidenced by positive values, except for BJLE, GNR, and Bitcoin, which display a leftward skew. All return series clearly show noticeable leptokurtosis features. The Jarque-Bera test statistics are significant, indicating that the daily returns are not normally distributed. The outcomes of the Ljung-Box test indicate the absence of autocorrelation in returns for ICLN, FAN, and Gold, as well as squared returns for all variables except CNRG and Bitcoin. This suggests the possible presence of volatility clustering in each of these return series. All these issues are considered by using different econometric models. The Lagrange multiplier test shows the presence of an ARCH effect among all return series, allowing us to use the ARCH model to capture the aforementioned stylized facts.

**Fig 1:**
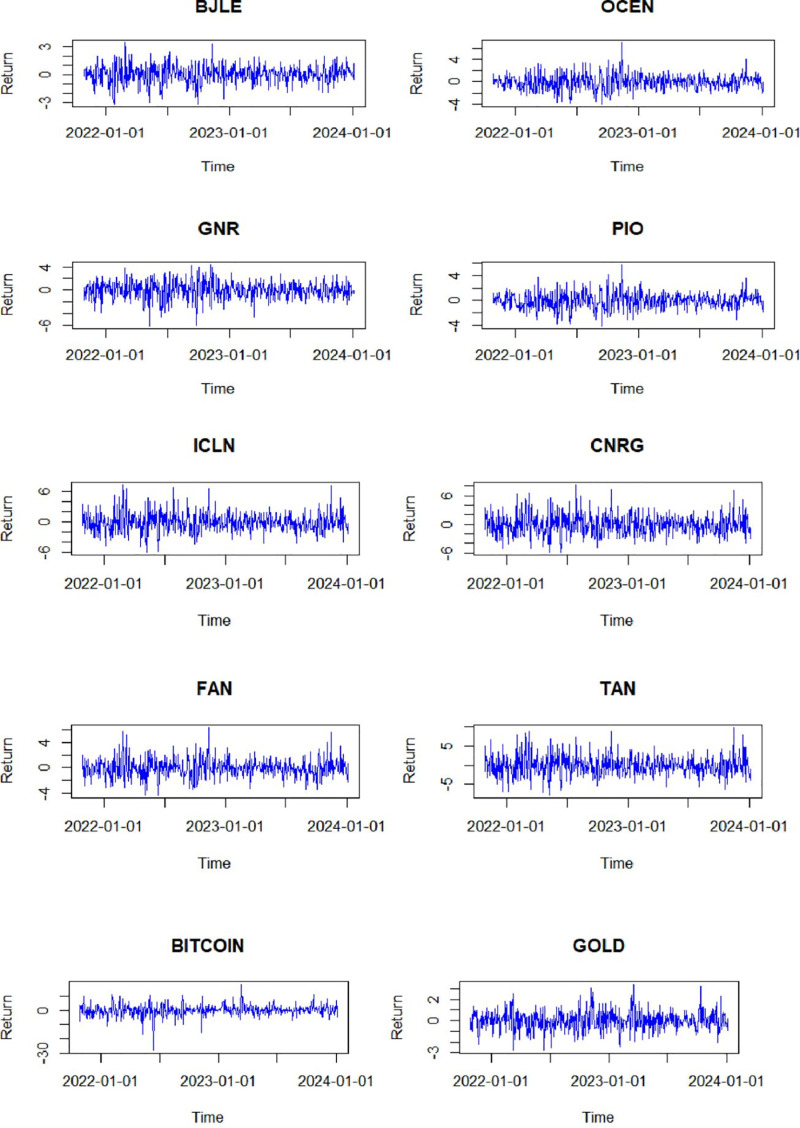
Return evolution of asset.

Fig 1 depicts the daily fluctuations in returns from October 25, 2021, to January 5, 2024. Different increasing and decreasing market phases are clearly documented for all time series. Most notably, significant declines are evident in all return series (except for Bitcoin), particularly with the onset of the Russia-Ukraine conflict in early 2022.

## 5. Empirical results

### 5.1. Diversification, hedging, and safe-haven features of bitcoin and gold against the blue economy and green assets

Table 3 provides valuable information regarding the diversification, hedging, and safe-haven characteristics of Bitcoin and gold against blue and green assets. From Table 3, it is evident that the DCC and ADCC models seem to yield approximately the same empirical results. The findings show that both Bitcoin and gold serve as diversifiers against all blue and green assets during stable periods. During periods of crisis, the correlation between Bitcoin and the different blue economy/green assets is negative and significant only for BJLE (at the 1% level) and OCEN (at the 10% level). This indicates the strong safe-haven ability of Bitcoin against such blue economy assets (BJLE and OCEN). On the other hand, a positive and significant correlation is observed only between the Bitcoin-BJLE pair, indicating the substantial diversifying role of Bitcoin against this blue economy asset at the 5% level. For the other assets, the correlation is either insignificantly negative or positive, suggesting the weak safe-haven and diversifying roles of Bitcoin for the remaining assets.

**Table 3:** Lucey equation results.

	DCC					
	[Intercept]	X1	X2	X3	R^2^	F^C^
**BTC/BJLE**	0,1863***	−0,0524***	0,0236***	0,0061	0,03534	6,644***
**BTC/OCEN**	0,3529***	0,0219	0,0027	−0,0513 *	0,03216	6,025***
**BTC/GNR**	0,2932***	0,0001	0,0114	−0,0277	0,01503	2,768**
**BTC/PIO**	0,3223***	−0,0050	−0,0088	−0,0133	0,03432	7,445***
**BTC/CNRG**	0,3495***	−0,0092	0,0113	−0,0103	0,00759	1,388
**BTC/ICLN**	0,3447***	−0,0155	0,0094	−0,0012	0,00673	1,229
**BTC/TAN**	0,3236***	−0,0055	−0,0030	−0,0017	0,00586	1,069
**BTC/FAN**	0,3210***	−0,0138	0,0053	0,0073	0,00308	0,5607
**GOLD/BJLE**	0,0863***	−0,1069***	0,0341**	0,0479 *	0,02017	3,733**
**GOLD/OCEN**	0,2060***	−0,0676	−0,0178	0,0780	0,00590	1,077
**GOLD/GNR**	0,3479***	−0,0688	−0,0098	0,0715	0,00481	0,8763
**GOLD/PIO**	0,2147***	0,0140	−0,0223	−0,0063	0,00337	0,6125
**GOLD/CNRG**	0,2395***	−0,0045	0,0079	−0,0110	0,00396	0,7209
**GOLD/ICLN**	0,1923***	0,0437	0,0004	−0,0540	0,00816	1,491
**GOLD/TAN**	0,3235***	−0,0168	−0,0260	0,0299	0,00628	1,147
**GOLD/FAN**	0,1831***	0,0121	0,0148 *	−0,0325**	0,01053	1,93
	**ADC**					
**BTC/BJLE**	0,1863***	−0,0524***	0,0236***	0,0061	0,03534	6,644***
**BTC/OCEN**	0,3677***	0,0204	0,0016	−0,0485 *	0,03547	6,669***
**BTC/GNR**	0,2932***	0,0001	0,0114	−0,0277	0,01503	2,768**
**BTC/PIO**	0,3415***	−0,0047	−0,0104	−0,0105	0,03878	7,317***
**BTC/CNRG**	0,3495***	−0,0092	0,0113	−0,0103	0,00759	1,388
**BTC/ICLN**	0,3447***	−0,0155	0,0094	−0,0012	0,00673	1,229
**BTC/TAN**	0,3236***	−0,0055	−0,0030	−0,0017	0,00586	1,069
**BTC/FAN**	0,3233***	−0,0139	0,0052	0,0073	0,00316	0,5752
**GOLD/BJLE**	0,0863***	−0,1069***	0,0341**	0,0479 *	0,02017	3,733**
**GOLD/OCEN**	0,2060***	−0,0676	−0,0178	0,0780	0,00590	1,077
**GOLD/GNR**	0,3479***	−0,0688	−0,0098	0,0715	0,00481	0,8763
**GOLD/PIO**	0,2147***	0,0140	−0,0223	−0,0063	0,00337	0,6125
**GOLD/CNRG**	0,2395***	−0,0045	0,0079	−0,0110	0,00396	0,7209
**GOLD/ICLN**	0,1923***	0,0437	0,0004	−0,0540	0,00816	1,491
**GOLD/TAN**	0,3235***	−0,0168	−0,0260	0,0299	0,00628	1,147
**GOLD/FAN**	0,1831***	0,0121	0,0148 *	−0,0325**	0,01053	1,93
	**GOGARCH**					
**BTC/BJLE**	0,2354***	0,0066	−0,0001	−0,0055	0,00239	0,4345
**BTC/OCEN**	0,4095***	0,0026	−0,0011	−0,0014	0,00199	0,3631
**BTC/GNR**	0,3072***	−0,0001	0,0027	−0,0104	0,01962	3,629**
**BTC/PIO**	0,3625***	−0,0021	0,0008	0,0030*	0,00723	1,32
**BTC/CNRG**	0,3709***	−0,0026	0,0009	0,0015	0,00296	0,538
**BTC/ICLN**	0,3753***	−0,0025	0,0007	0,0016	0,00057	0,1039
**BTC/TAN**	0,3444***	0,0031	0,0072	−0,0074	0,00772	1,411
**BTC/FAN**	0,3517***	−0,0002	0,0006	−0,0001	0,00084	0,152
**GOLD/BJLE**	0,0732***	−0,1633***	0,0542**	0,0810**	0,01920	3,55**
**GOLD/OCEN**	0,2091***	−0,0650	−0,0136	0,0644	0,00558	1,018
**GOLD/GNR**	0,3428***	−0,0538	−0,0031	0,0502	0,00698	1,274
**GOLD/PIO**	0,2152***	0,0205	−0,0275	−0,0151	0,00495	0,9013
**GOLD/CNRG**	0,2382***	−0,0060	−0,0018	−0,0039	0,00690	1,26
**GOLD/ICLN**	0,1942***	0,0270	−0,0047	−0,0333	0,00753	1,375
**GOLD/TAN**	0,3216***	−0,0128	−0,0292	0,0218	0,00969	1,775
**GOLD/FAN**	0,1799***	0,0109	0,0100	−0,0271	0,00727	1,328

Note: R^2^ and F^C^ design the Multiple R-squared and Fisher statistic test respectively.

Turning to gold, the correlation between the yellow metal and the different blue economy/green finance assets is negative and significant only for the blue economy asset BJLE (at the 1% level) and the green finance asset FAN (at the 5% level). This indicates the strong safe-haven ability of gold against such blue economy/green finance assets. Moreover, positive and significant correlations are observed between the gold-BJLE pair at the 5% and 10% levels and between the gold-FAN pair at the 10% level, indicating the substantial diversifying ability of gold against these assets. For the other pairs, the correlation seems to be insignificantly negative or positive, suggesting the weak safe-haven and diversifying roles of gold.

Based on the GO-GARCH estimation results, it is evident that Bitcoin and gold play the role of strong diversifiers against all the blue economy and green finance assets during normal times. However, gold tends to be a robust safe haven only against BJLE at the 1% level and a strong diversifier against this blue economy asset at the 5% and 10% levels. On the other hand, Bitcoin acts only as a strong diversifier for PIO.

Based on the aforementioned results, it is worth noting to mention that gold outperforms Bitcoin given that gold plays simultaneously the role of a diversifier and a safe-haven asset during turbulent times. This emphasizes its potential as a comparatively stable and protective asset in comparison with Bitcoin. These findings corroborate those of [6,46,47] who show that gold outperforms Bitcoin as a safe-haven asset during turbulent times.

From Table 3, all R-squared are low (close to 0) indicating that the dummy variables do not explain much of the variability of the correlation using the three models. Based on the F-statistic, only for BTC/BJLE, BTC/OCEN, BTC/GNR, BTC/PIO and GOLD/BJLE (when using DCC and ADCC models) and BTC/GNR and GOLD/BJLE (when using GOGARCH model) indicate that the model is statistically significant. This means that at least one of the independent variables is significantly related to the correlation variable.

### 5.2. Hedge ratios (HR) and hedging effectiveness (HE) results

The estimation findings for hedge ratios (HR) and hedging effectiveness (HE) under the DCC, ADCC, and GO-GARCH models are presented in Table 3. From Table 4, it is worthwhile that the DCC and ADCC models consistently produce similar results. As well, the GO-GARCH model consistently shows the highest value of hedge effectiveness index across all the pairs of assets. Under the GO-GARCH specification, the portfolio seems to be more effective in reducing risk compared to the DCC and ADCC models. These findings corroborate those of [32], who found that the GO-GARCH model provides the best value of HEs’ index compared to the DCC and ADCC models. In this regard, the HE estimation results based on the GO-GARCH model show that gold is the best hedging instrument only for GNR and TAN whereas Bitcoin is considered as the best hedging instrument for the rest of the blue economy and green finance assets given their higher value of HE index. These findings contradict the outcomes of [40,66], who show that gold outperforms Bitcoin as a hedging instrument. However, it corroborates those of [99] who generally support the strong hedging ability of cryptocurrencies. On the other hand, the same results are recorded when computing the HE index using the DCC and ADCC models.

**Table 4:** Hedging ratio and hedging effectiveness.

		DCC		ADCC		GOGARCH	
		BETA	HE	BETA	HE	BETA	HE
**BITCOIN**	**BJLE**	0,0490	0,0457	0,0490	0,0457	0,2368	0,0559
	**OCEN**	0,1246	0,1479	0,1292	0,1571	0,4128	0,1675
	**GNR**	0,1149	0,1062	0,1149	0,1062	0,3158	0,1000
	**PIO**	0,1072	0,1197	0,1125	0,1308	0,3669	0,1319
	**CNRG**	0,1713	0,1395	0,1713	0,1395	0,3717	0,1381
	**ICLN**	0,1924	0,1373	0,1924	0,1373	0,3812	0,1416
	**TAN**	0,1197	0,1168	0,1197	0,1168	0,3452	0,1204
	**FAN**	0,2115	0,1205	0,2131	0,1218	0,3534	0,1241
**GOLD**	**BJLE**	0,1211	0,0298	0,1211	0,0298	0,0601	0,0508
	**OCEN**	0,3487	0,1151	0,3487	0,1151	0,3277	0,1210
	**GNR**	0,5922	0,1542	0,5922	0,1542	0,2847	0,1706
	**PIO**	0,3302	0,1130	0,3302	0,1130	0,3257	0,1281
	**CNRG**	0,5397	0,0774	0,5397	0,0774	0,2789	0,0814
	**ICLN**	0,5073	0,0628	0,5073	0,0628	0,1661	0,0717
	**TAN**	0,5368	0,1329	0,5368	0,1329	0,3858	0,1414
	**FAN**	0,5808	0,0519	0,5808	0,0519	0,1653	0,0539

As far as the hedging ratio (HR) is concerned, a negative value of HR indicates that investors should adopt the same position [either short or long] for two assets within the same portfolio. Nevertheless, a positive value of HR suggests that inverse position is required to hedge against the risk associated to each asset. Investors should take inverse positions to hedge against the risk associated with different blue economy and green finance assets under different models. For instance, concerning the DCC model, a $1 long position in BJLE can be hedged by a short position of 0.0490 cents in Bitcoin or with a short position of 0.1211 cents in gold during turbulent periods.

Moreover, a lower hedge ratio implies a more cost-effective hedge [100]. Using the DCC and ADCC models, the hedge ratios for Bitcoin are lower compared to those for gold. Such findings suggest that a considerably greater amount ($) is required for the efficient hedging of blue economy and green finance assets through gold. However, based on the GO-GARCH model, we show that gold is more effective than Bitcoin. This could be explained by the fact that the GO-GARCH model takes into consideration the volatility spillover when estimating the GO-GARCH model. Such feature seems to be absent in the DCC and ADCC models [32].

### 5.3. Discussion

Our study investigates the diversification, hedging, and safe-haven characteristics of Bitcoin and gold against blue economy and green finance assets, using DCC, ADCC, and GO-GARCH models. The findings highlight significant empirical distinctions between Bitcoin and gold in their roles within diversified portfolios.

Bitcoin serves as a diversifier during stable periods across all evaluated assets, with notable safe-haven attributes observed during crises against BJLE and OCEN. These findings suggest Bitcoin’s potential as a protective asset during market turmoil, albeit with varied effectiveness across different assets. In contrast, gold demonstrates robust safe-haven properties against BJLE and FAN, along with substantial diversifying abilities against these assets, underscoring its stability and attractiveness during economic uncertainties.

The GO-GARCH model consistently shows superior hedging effectiveness indices compared to DCC and ADCC models, emphasizing its efficacy in risk reduction across asset pairs. Gold emerges as the most effective hedging instrument for GNR and TAN, whereas Bitcoin proves optimal for other blue economy and green finance assets, aligning with previous studies highlighting cryptocurrencies’ strong hedging abilities [82,85,86,90,99].

Hedge ratios further illustrate practical insights for investors, suggesting appropriate portfolio adjustments during turbulent periods. Bitcoin generally exhibits lower hedge ratios than gold under DCC and ADCC models, indicating cost-effective hedging potential. Conversely, the GO-GARCH model identifies gold’s superior effectiveness due to its consideration of volatility spillover effects, offering nuanced strategies for risk management in diverse market conditions.

To sum up, our findings contribute valuable insights into the roles of Bitcoin and gold as diversifiers, safe havens, and hedging instruments within portfolios containing blue economy and green finance assets. These results inform investors and policymakers seeking to optimize portfolio resilience during evolving market dynamics and environmental considerations.

## 6. Conclusion

Following [28], we aim to explore the diversifying, hedging, and safe haven abilities of Bitcoin and gold against blue economy and green assets using three different MGARCH models (DCC, ADCC, and GO-GARCH). The regression analyses reveal that both the DCC and ADCC models generate similar results.

During stable periods, Bitcoin and gold act as effective diversifiers, exhibiting positive correlations with all blue and green assets. In times of crisis, Bitcoin demonstrates a robust safe-haven ability, particularly against the blue economy assets BJLE and OCEN. Furthermore, the correlation analysis with gold unveils its pronounced safe-haven ability against specific blue economy and green finance assets, such as BJLE and FAN. Positive and significant correlations between gold and BJLE, as well as gold and FAN, underscore gold’s substantial diversifying role against these assets. However, the correlation is either insignificantly negative or positive for other pairs, indicating a comparatively weaker safe haven and diversifying influence of gold. Turning to the GO-GARCH estimation results, both Bitcoin and gold emerge as strong diversifiers during normal times. Notably, gold exhibits a robust safe-haven role against BJLE at the 1% level and serves as a strong diversifier against this blue economy asset at the 5% and 10% levels. Conversely, Bitcoin functions primarily as a strong diversifier for PIO. Considering these outcomes, it is pertinent to highlight that gold surpasses Bitcoin in terms of its dual role as a diversifier and a safe-haven asset during turbulent times.

As far as hedging effectiveness is concerned, the GO-GARCH model consistently outperforms the DCC and ADCC models in reducing risk. The GO-GARCH model shows that gold acts as the best hedging instrument for GNR and TAN, while Bitcoin tends to be more effective for other blue and green assets. The hedging ratios indicate that investors should take inverse positions to hedge against risks associated with different assets, irrespective of the model used. Notably, the GO-GARCH model suggests that hedging with gold is more cost-effective than with Bitcoin, given that the GO-GARCH model takes into account the volatility spillover compared to the DCC and ADCC models.

The findings of this study offer several practical implications for investors and policymakers. During stable market conditions, both Bitcoin and gold can serve as effective diversification tools when constructing portfolios that include blue economy and green finance assets, suggesting that investors may consider allocating a portion of their portfolios to these precious metals to mitigate overall risk exposure. In times of market turbulence or economic uncertainty, gold emerges as a robust safe haven, particularly against specific blue economy assets like BJLE and green finance assets like FAN, highlighting its role in preserving capital and providing stability during crises. The differential hedging effectiveness between Bitcoin and gold underscores their distinct roles in risk management strategies, with Bitcoin offering strong diversification benefits across various assets and gold’s superior hedging effectiveness, especially under the GO-GARCH model, suggesting it as a preferred choice for reducing portfolio volatility and enhancing risk-adjusted returns. Policymakers should consider issuing guidelines for institutional investors regarding the inclusion of gold and Bitcoin in portfolios to achieve optimal diversification and hedging outcomes, promoting systemic stability and sustainable investments.

While this study provides valuable insights during periods of significant global disruptions, it is not without limitations. Firstly, the analysis is constrained by the availability of data, which is only available from October 25, 2021, limiting the inclusion of a non-crisis reference period. This restricts our ability to compare asset performance during stable times versus crisis periods. Secondly, the study primarily relies on daily closing prices, which may not capture intraday volatility and market nuances. Future research could benefit from incorporating high-frequency data to provide a more granular analysis of asset performance. Additionally, expanding the dataset to include a broader range of assets and geographic regions could enhance the robustness and generalizability of the findings. Finally, while the ARCH model was employed to account for volatility clustering, exploring other advanced econometric models could provide deeper insights into the dynamics of asset returns under adverse conditions. Addressing these limitations in future research could further strengthen the understanding of sustainable investments in the blue and green economy sectors.
